# Caregivers' knowledge, practice, and associated factors toward oral rehydration salt with zinc to treat diarrhea among under 5 children in Burayu town, Oromia, Ethiopia, 2022: Cross‐sectional study: An implication for action

**DOI:** 10.1002/hsr2.1817

**Published:** 2024-01-22

**Authors:** Nadhi A. Duguma, Elias Teferi Bala, Bayisa Abdisa, Timketa Adula, Emiru Adeba, Gudina Egata

**Affiliations:** ^1^ Oromia Health Bureau Finfine Ethiopia Finfine Ethiopia; ^2^ Department of Public Health Ambo University Ambo Ethiopia; ^3^ Department of Public Health Wollega University Nekemte Ethiopia; ^4^ Department of Nutrition and Dietetics Addis Abeba University Finfine Ethiopia

**Keywords:** caregivers, diarrhea, knowledge, ORS, practice, zinc

## Abstract

**Background:**

Diarrhea is the second leading cause of death in under 5 children of Ethiopia. Millions of young lives could be saved if mothers know and practice the three rules of diarrhea management: giving extra fluid (particularly oral rehydration salt [ORS]), zinc, and giving additional food during diarrhea illness.

**Aim:**

The objective of this study was to determine mothers'/caregivers' Knowledge, Practice, and associated factors toward ORS with zinc to treat diarrhea among under 5 children in Burayu town, Oromia Region, Ethiopia, 2022.

**Methods:**

A community‐based cross‐sectional study was conducted among 422 study participants from September 25 to October 10, 2022; in Burayu town, Ethiopia. Systematic random sampling technique was used to enroll study subjects from two randomly selected kebeles. Interviewer‐administered structured questionnaire was used to collect data. Data were entered into Epi‐info version 3.5.1 and exported to SPSS Version 21 for analysis. Binary and multivariable logistic regression was done to identify factors associated with knowledge and practice of ORS with zinc at *p* < 0.05% and 95% confidence interval (CI).

**Results:**

The findings showed that 59% and 52% of the respondents had good knowledge and practice toward ORS with zinc, respectively. Being housewives (adjusted odds ratio, AOR = 0.407, 95% CI: [0.195, 0.848]), primary education (AOR = 3.246, 95% CI: [1.614, 6.530]), income of >4000 (AOR = 5.132, 95% CI: [1.947, 13.524]), health seeking behavior (AOR = 0.369, 95% CI: [0.139, 0.979]), being divorced (AOR = 0.275, 95% CI: [0.09,0.842]) were found to be significantly associated with knowledge toward management of diarrhea with ORS and zinc. Housewives in occupation (AOR = 0.084, 95% CI: [0.029, 0.243]), secondary and above education (AOR = 6.26: 95% CI: [1.51, 25.86]), health seeking behavior (AOR = 6.885, 95% CI: [2.29, 20.67]), having good knowledge of ORS and zinc (AOR = 22.14, 95% CI: [8.44, 58.07]) were found to be significantly associated with practice of managing diarrhea with ORS and zinc.

**Conclusion:**

This study revealed low level of knowledge and practice of caregivers toward ORS with zinc. The more mothers/caregivers are knowledgeable, the more they are active to practice the use of ORS with zinc. Thus, health education and awareness creation for the mothers/caregivers on management of diarrhea is very crucial. Special attention should be given to enhancing income for mothers/caregivers.

## 1 BACKGROUND

World Health Organization (WHO) defines diarrhea as the passage of three or more loose or watery stools in 24‐h duration.^1^ Diarrhea is still among the major causes of mortality among children under 5 years of age; by making the second leading cause of death. About 437,000 of under 5 children are dying of diarrhea each year worldwide.^2^ Majority of these deaths are as result of dehydration and mismanagement. Diarrhea is both preventable and treatable disease, but due to improper knowledge of mothers and their misdirected approach toward the disease management results in severe dehydration and death.^3^

The goals of diarrhea treatment are to maintain or recover hydration, treat the underlying causes, and relieve symptoms. The use of oral rehydration salt (ORS) is extremely effective in treating acute watery diarrhea and has markedly contributed to reducing childhood deaths. Currently, many diarrhea‐associated deaths could be prevented by timely treatment with ORS.^4^ Timely management of children with ORS and zinc has substantially declined the mortality and morbidity from acute diarrhea.

Giving ORS after each loose stool until diarrhea stops and supplementing zinc for 10–14 days are safe and effective in both home and facility settings when properly prepared and administered. However, awareness of under 5 caregivers on zinc supplementation is low; consequently its utilization is limited.^3^, ^5^, ^6^, ^7^, ^8^ In addition, there is knowledge gap among health care providers about zinc supplementation for diarrheal treatment.^9^ More than 60% of childhood diarrhea could be prevented with full coverage of ORS and zinc.^1^ Yet, of those children with acute diarrhea, only a median of 42% receive ORS and less than 7% receive both ORS and zinc globally.^10^ Zinc supplements reduce the duration of diarrheal episodes by 25% and reduce stool volume by 30%.^1^, ^5^, ^11^ It is also a vital micronutrient essential for protein synthesis, cell growth and differentiation, immune function, and intestinal transport of water and electrolytes.^12^

Childhood diarrhea accounts for 10% of all causes of morbidity in under 5 children of Ethiopia and it is the second leading cause of deaths, after pneumonia.^13^, ^14^ More than three‐quarters of all diarrhea deaths could be averted with full coverage and utilization of ORS and zinc supplementation.^15^

Ethiopia has been using different strategies and has made many efforts to reduce diarrhea related morbidity and mortality. Integrated Management of Childhood Illness is one of the strategies which focuses on the management of the top 5 diseases among under 5 children including diarrhea. Accordingly, every child with diarrhea should be treated with ORS and zinc.^16^ However, utilization of ORS and zinc is influenced by many factors such as caregivers' sociodemographic status, socioeconomic status, previous experience, and other factors. Mothers' knowledge, attitude, and practice about ORS and zinc are the main determinant of diarrhea management.^6^, ^17^, ^18^, ^19^, ^20^

On the other hand, research on the knowledge and practice toward zinc and ORS among mothers' of children with diarrheal diseases is scarce in Ethiopia. To assess the success of zinc supplementation along with ORS in diarrheal diseases management, and to find ways of promoting it, a study on caregivers' knowledge and practice toward zinc with ORS and associated factors to treat children with diarrhea was needed. Therefore, in this study knowledge, practice, and associated factors toward ORS with zinc among mothers/caregivers of under 5 children were assessed.

## 2 METHODS

### 2.1 Study area and period

The study was conducted in Burayu town. Burayu town is located in the western fringe of Addis Ababa the Capital city of Ethiopia. The town has 375,349 total populations, about 50,513 households (HHs) and 61,670 under 5 children.^21^ Due to this demographic pressure water scarcity is common in the town. The current water supply coverage of the city is 60% which can contribute to diarrheal diseases in the town. Burayu town has six kebeles. The town has four health centers, 51 medium clinics, two small clinics, 11 pharmacies, 44 drug stores, and six health posts.^22^ The study was conducted from September 25 to October 10, 2022.

### 2.2 Study design

A community‐based cross‐sectional study was conducted in Burayu town.

### 2.3 Population

Source population: All mothers/caregivers having under 5 children and living in Burayu town.

#### 2.3.1 Study population

All mothers/caregivers having under 5 children in randomly selected kebeles, in Burayu town.

#### 2.3.2 Sampling unit

HHs having under 5 children.

#### 2.3.3 Study unit

Care giver/mother having children younger than 5 years.

#### 2.3.4 Inclusion criteria

Mothers/caregivers who have under 5 children and have willing to participate in the study in selected sub kebeles during the study period.

#### 2.3.5 Exclusion criteria

None of the mothers/caregivers under sampling plan were not fulfilled the exclusion criteria.

### 2.4 Sample size determination and sampling technique

The sample size was determined by using a single population proportion formula with the following assumptions; estimation of the proportion of knowledge of ORS with zinc in the study area as 50%, 95% confidence interval (CI), 5% margin of error (*d*), and 10% nonresponse rate.

$$\text{Sample}\;\text{size}\;(n)=\frac{\left[Z{\left(\alpha /2\right)}^{2}\;\times \;p(1‐p)\right]}{d^{2}},$$

where *z* is the standard normal distribution (1.96) at 95% CI, *p* is the proportion of knowledge of ORS with zinc in the study area (50%), *d* is the margin of error (5%) n= required sample size

$$n=\frac{\left[{\left(1.96\right)}^{2}\times 0.5(1-0.5)\right]}{\;{\left(0.05\right)}^{2}},$$

*n* = 384, by adding 10% of nonresponse rate, the total sample size became 422 of under 5 years children's mothers/caregivers.

### 2.5 Sampling technique

**Figure 1:**
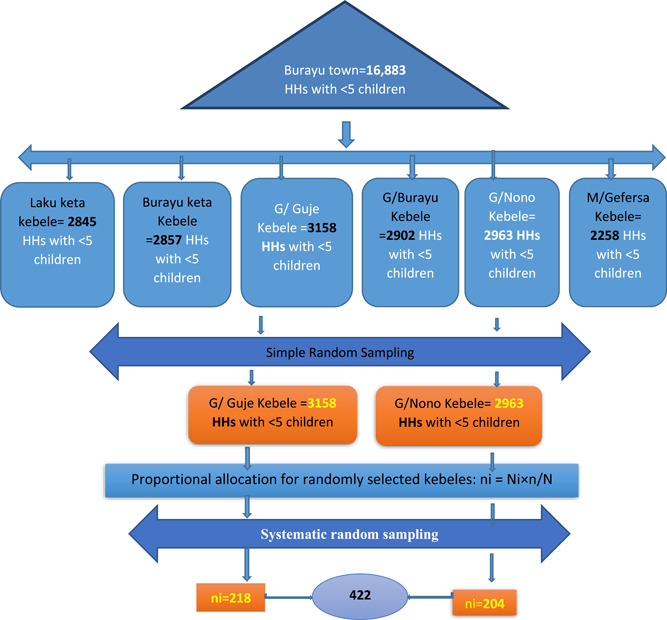
Sampling procedure, to assess caregivers' knowledge and practice toward oral rehydration salt with zinc and associated factors to treat diarrhea in under 5 children of Burayu Town, Oromia, Ethiopia, 2022.

The list of registrations of HHs who had under 5 children was taken from local health extension workers of the kebeles; two kebeles were selected randomly from six kebeles in Burayu town. Accordingly, the total number of HHs who had under 5 children in the two selected kebeles were 6121. The number of mothers who have under 5 children was selected from the two kebeles (Gafersa Guje and Gafersa Nono) based on the population proportion size of the study population. Study HHs were selected by systematic random sampling method every 15 (Figure 1).

### 2.6 Study variables

#### 2.6.1 Dependent variable

Knowledge of mothers/caregivers toward ORS with zinc supplementation.

Practice of mothers/caregivers toward ORS with zinc supplementation.

#### 2.6.2 Independent variables

##### Sociodemographic status

Age of respondent, sex of respondent, marital status, educational status, religion, ethnicity, occupation, Relation of caregivers to a child, income.

##### Maternal related factors

Mothers'/caregivers' health seeking behavior, place sought for treatment/type of health facility visited, previous experience (ever experienced child with diarrhea).

##### Health facility‐related factors

Availability of ORS with zinc, price of ORS with zinc.

### 2.7 Operational definition

#### 2.7.1 Good knowledge

Those mothers who answered correctly above the mean of the knowledge questions were considered as having good knowledge.^17^, ^19^, ^23^, ^24^, ^25^

#### 2.7.2 Poor knowledge

Those mothers who answered below the mean of the knowledge assessing questions were considered as having poor knowledge.

#### 2.7.3 Good practice

Those mothers who able to answer above the mean of the practice assessing questions were considered as having good practice.^17^, ^19^, ^23^, ^24^, ^25^

#### 2.7.4 Poor practice

Those mothers who answer below the mean of the practice assessing questions were considered as having poor practice.

#### 2.7.5 Caregiver

Who directly give care for child and bring to health facility or other else during illness.

#### 2.7.6 ORS with zinc

A combination of oral rehydration salt and zinc supplement which could available either separately or packed together (two sackets of ORS with 10 tabs of zinc).

### 2.8 Data collection tools and techniques

Data were collected by using pretested, interviewer‐administered structured questionnaires. The questionnaires were developed in English after reviewing of related available literature.^17^, ^19^, ^23^, ^25^, ^26^ The English version of the questionnaire was translated to regional languages of the community (Afan Oromo and Amharic). The questionnaire had three parts: (sociodemographic characteristics of caregivers, knowledge of caregivers toward ORS with zinc supplementation, and practice of caregivers toward ORS with zinc supplementation to treat diarrhea in under 5 children). Questions on practice were asked only mothers/caregivers whose child ever experience diarrhea. Data were collected by four BSc nurses who were fluent in both Afan Oromo and Amharic languages. During data collection, where more than one under 5 children in the selected household, only one child was selected by lottery method.

### 2.9 Data quality control and management

First, data collectors were received training before the data collection. The prepared sets of questionnaires were pretested 1 week before data collection on 21 (5% sample size) of under 5 children's mothers/caregivers in Gefersa Burayu Kebele who were not included in the main study; to maintain accuracy and clarity of the questionnaire and to check the consistency in interpretation. Questionnaires were revised in accordance with the findings of pretesting and all the ambiguous, misleading, and wrongly interpreted questions were omitted. Questions on practice were asked depending on last/recent episode of diarrhea to minimize recall bias. Questionnaires were also checked daily for completeness, consistency, and clarity by the researcher. In addition, the thesis supervisors (two health officers) and the researcher were also visited the data collection sites periodically to monitor the process of data collection.

### 2.10 Data processing and analysis

First, the data were checked for completeness and consistency. Then, it was coded, cleaned; and entered by using epi‐info software version 3.1 and then, exported to SPSS version 21 for analysis. Descriptive statistics was used to summarize the sociodemographic characteristics of the study participants, level of knowledge, and practice for knowledge and practice. Tables, charts, and graphs were used to present data frequencies and percentages. The level of knowledge and practice were dichotomized as “Good” or “Poor” depending on the mean after the normality of data distribution was checked by histogram. Bivariate and multivariate logistic regression analysis was employed to examine the associations between independent variables and the outcome variables. First, bivariate logistic regression was performed to each independent variable with the outcome variable; and variables with *p* < 0.25 were made candidate for the multivariable analysis. Multicollinearity was checked by collinearity matrix, tolerance test, and variance inflation factor. Model fitness was also checked with Hosmer–Lemeshow goodness‐of‐fit test. Multivariate logistic regression analysis was computed to identify associated factors with the knowledge and practice of mothers/caregivers. Strength of association was measured using adjusted odds ratio, at 95% CIs. Statistical significance was declared at *p* < 0.05.

### 2.11 Ethical and legal considerations

Ethical clearance was obtained from the Institutional Review Board of Ambo University, College of Medicine and Health Sciences. Official permission letter was given to the Burayu town Health office. Then Burayu town Health office sent official support letter to selected kebeles. Verbal informed consent from parents/caregivers of under 5 children was obtained and each study participant was adequately informed about the objective of the study and anticipated benefit and risk of the study by their data collectors. Privacy and confidentiality of collected information was ensured at all level. Respondents were also told the right not to respond to the questions if they do not want to respond or to terminate the interview at any time.

## 3 RESULTS

### 3.1 Sociodemographic characteristics of respondents

A total of 422 respondents participated in the study yielding a response rate of 100%. Of the total participants, 230 (54.5%) were in the age range of 25–34 years with mean (+SD) age of 27.9 ± 5.3 years. More than nine out of 10 (91.9%) of the study participants were married. More than half, 224 (53.1%) of the study participants were Oromo followed by silte 73 (17.3%) ethnic group.

One hundred and fifty (35.5%) of the study participants were Orthodox Christian followed by protestant 31.3% and 30.6% of respondents were Muslim by religion. The mean monthly income was 2700 and SD ± 2587. Concerning occupational status 182 (43.1%) of the participants were housewives followed by 107 (25.4%) employees (government, private, NGOs), merchant 65 (25.36%). Regarding the educational status of respondents 111(26.3%) had no formal education, 107 (25.4%) of them had primary education, and 140 (33.27%) had above secondary education (Table 1).

**Table 1:** Sociodemographic characteristics of caregivers of under 5 children in Burayu town, Oromia, Ethiopia, 2022.

Variables	Response	Frequency	%
Age category	<25	148	35.1
	25–34	230	54.5
	≥35	44	10.4
Sex of respondent	Male	45	10.7
	Female	377	89.3
Relation to child	Mother	365	86.5
	Father	40	9.5
	Others^a^	17	4.0
Occupational status	Employee	107	25.36
	Housewife	182	43.13
	Merchant	65	15.4
	Farmer	32	7.6
	Daily laborer	16	3.71
	Student	20	4.8
Educational status	No formal education	111	26.3
	Primary education (1–8 grade)	107	25.36
	Secondary education (9–12 grade)	64	15.17
	Above secondary education	140	33.27
Religion	Orthodox	150	35.5
	Protestant	132	31.3
	Muslim	129	30.6
	Other^b^	11	2.6
Ethnicity	Oromo	224	53.1
	Amhara	47	11.1
	Silte	73	17.3
	Gurage	61	14.5
	Other^c^	17	4.0
Marital status	Married	388	91.9
	Widow	9	2.1
	Divorced	25	5.9
Income category^19^	≤2560 (lowest income)	269	63.7
	2561–3200 (second income)	47	11.1
	3201–4000 (middle income)	20	4.7
	>4000 (fourth income)	86	20.3

^a^ Grand mother, sister, relative.

^b^ Wakefata, adventist.

^c^ Tigre, Dorze, Gamo.

### 3.2 Knowledge of mothers/caregivers about ORS with zinc supplementation

Among respondents 403 (95.5%) heard about ORS. Among who heard about ORS 337 (83.6%) reported that ORS replaces lost fluid during diarrhea and 58 (14.4%) did not know the role of ORS. Three hundred seventy‐one (92.1%) caregivers mentioned that one liter of water is needed to dissolve a sachet of ORS and 261 (64.8%) reported that giving ORS should start as soon as diarrhea starts. Majority of the participants 333 (82%) know the duration of time when the prepared ORS can preserved (i.e., 24 h); and more than half 179 (55.6%) of respondents did not know when to stop giving ORS for child with diarrhea.

Regarding zinc supplementation 287 (68%) of study subjects heard about zinc supplementation. Among who heard about zinc 218 (76%) reported that zinc supplement shortens the duration of diarrhea/stops diarrhea and 114 (39.7%) know that zinc reduces severity of diarrhea, while 19 (6.6%) belief that zinc replaces lost fluid with diarrhea and 57 (19.9%) of them did not know the role of zinc in diarrhea management. About 180 (62.7%) also responded that zinc supplement can reduce episode of diarrhea and 118 (41.1%) reported that zinc supplementation during diarrhea increases the appetite of children. Majority 241 (84%) the study subjects know how often zinc should be given, while 136 (47.4%) individuals know the duration zinc should be given. Most of the participants 260 (90.6%) know how to administer zinc for children with diarrhea (Table 2).

**Table 2:** Knowledge of respondents about ORS with zinc supplementation for diarrhea management in Burayu town, Oromia, Ethiopia, 2022.

Variables	Response	Frequency	%
Heard about ORS (*n* = 422)	Yes	403	95.5
	No	19	4.5
Role of ORS (*n* = 403)	Replaces lost fluid due to diarrhea	337	83.6
	Shortens duration of diarrhea	4	1
	Reduce severity of diarrhea	4	1
	Don't know	58	14.4
Know preparation of ORS (*n* = 403)	Correct answer	371	92.1
	incorrect	32	7.9
when should giving ORS start	Correct answer	261	64.8
	Incorrect	142	35.2
Know quantity of ORS should be given	Correct answer	153	38.0
	incorrect	250	62.0
know duration ORS can preserved	Correct answer	333	82.6
	Incorrect	70	17.4
Know when to stop ORS	Correct answer	224	44.4
	incorrect	179	55.6
Know zinc supplementation	Yes	287	68.0
	No	135	32.0
Know role of zinc			
Shorten duration	Yes	218	76.0
	No	69	24.0
Reduce severity	Yes	114	39.7
	No	173	60.3
Replace lost fluid	Yes	19	6.6
	No	268	93.4
Don't know	Yes	57	19.9
	No	230	80.1
Zinc reduce episode (*n* = 287)	Yes	180	62.7
	No	107	37.3
Zinc increase appetite	Yes	118	41.1
	No	169	58.9
Know frequency zinc should be given	Correct answer	241	84.0
	incorrect	46	16.0
Know duration zinc should be given	Correct answer	136	47.4
	Incorrect	151	52.6
Know how to administer zinc tablet for child	Correct answer	260	90.6
	Incorrect	27	9.4

Abbreviation: ORS, oral rehydration salt.

**Figure 2:**
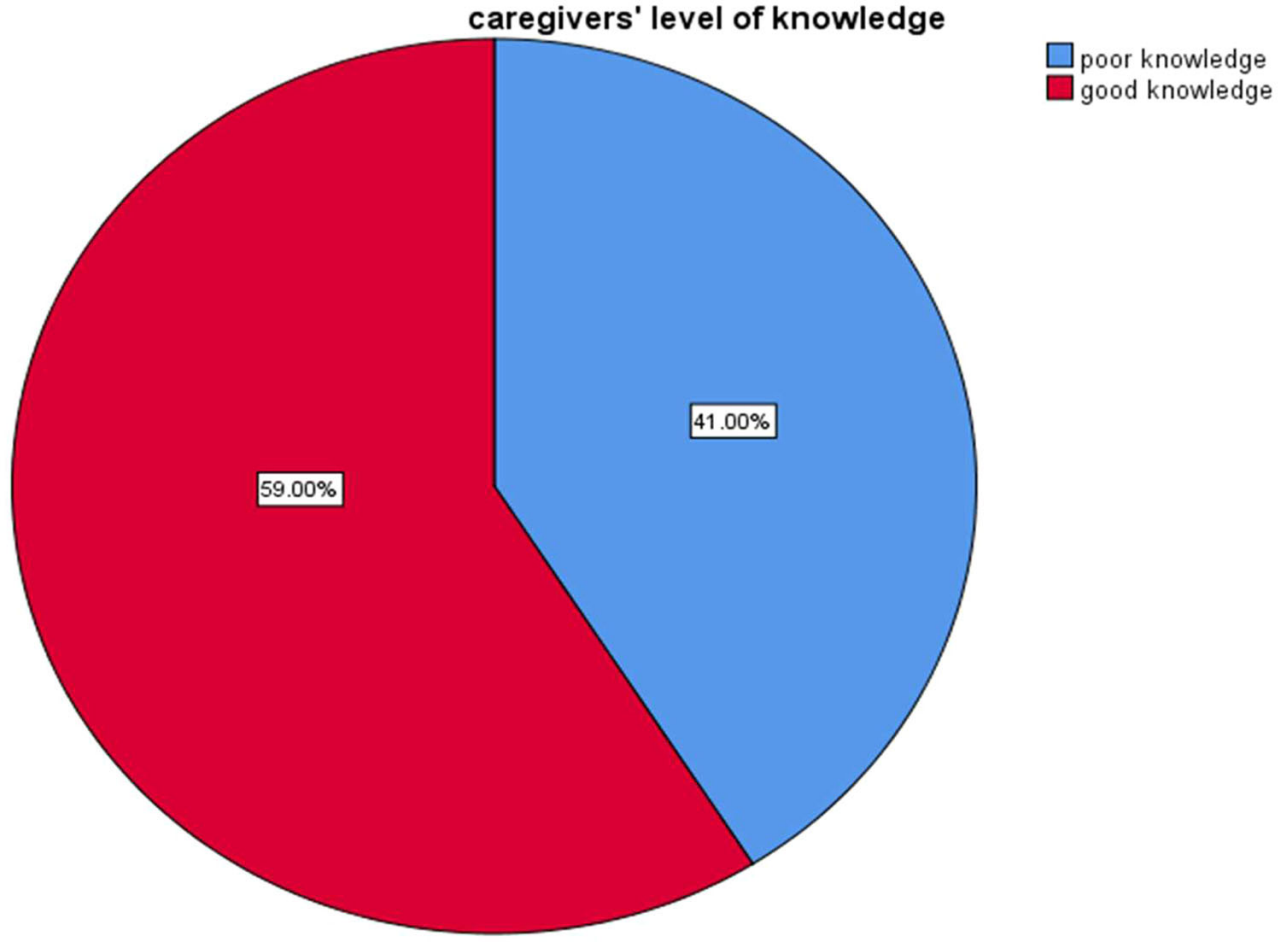
Level of knowledge of under 5 caregivers about oral rehydration salt with zinc in Burayu town, Oromia, Ethiopia, 2022.

Overall, 243 (59%) of mothers/caregivers had good knowledge of ORS with zinc depending on the mean of 16 questions asked to assess level of knowledge (Figure 2).

### 3.3 Practice of mothers/caregivers toward ORS with zinc in treatment of diarrhea

**Figure 3:**
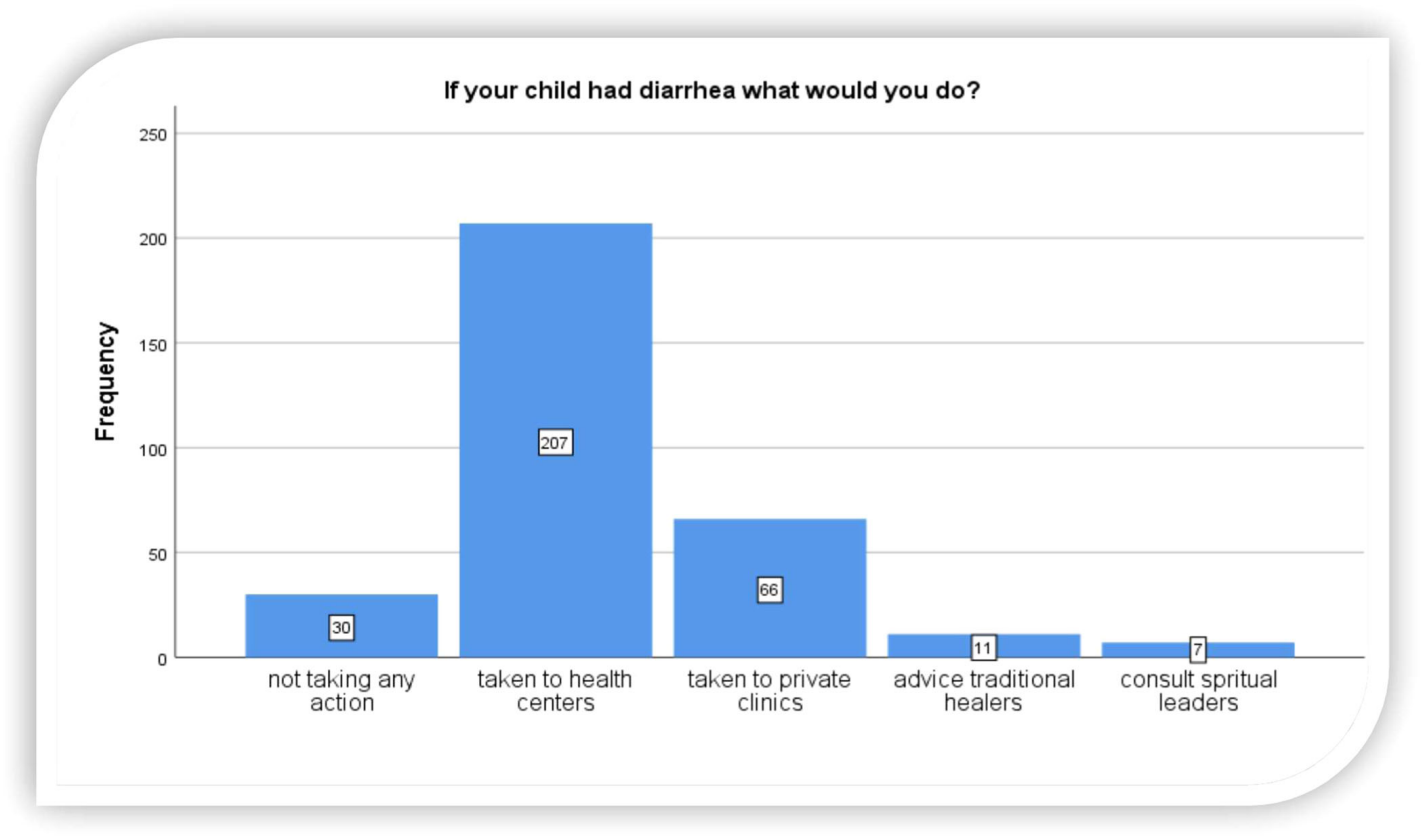
Health seeking behavior of child caregivers on diarrhea management in Burayu town, Oromia, Ethiopia, 2022.

Three hundred and twenty‐one (76.1%) of mothers ever cared for a child who had diarrhea. Majority 207 (64.5%) of these children were taken to government health facility (health centers), 66 (20.6%) were taken to private clinics. The rest 30 (9.3%) were waiting for spontaneous recovery without taking any action, 11 (3.4%) were taken to traditional healers and 7 (2.2%) of caregivers were consulted spiritual leaders and prayed for their children during diarrhea (Figure 3).

Among those children who had diarrhea 175 (54.5%) were given ORS with zinc supplementation while; 102 (31.8%) children were given only ORS, 109 (34%) given homemade fluid (soup, juice, rice water, and etc.), and 25 (7.8%) were given sugar–salt‐solution for their children with diarrhea infections. Eighty five (26.5%) of children were given antibiotics/antidiarrheal for diarrhea treatment.

A few of caregivers gave usual food and restrict fluid during diarrheal episode. When asked the reasons why they do not use ORS with zinc: 29 (20.1%) replied they did not know where to obtain it, 56 (38.9%) replied it is not available easily at that time, 59 (41%) mentioned they could not afford it. Majority 153 (55%) of the participants started ORS soon after noticed loose stool while, 72 (25.9%) started ORS after 1 day, 44 (15.8%) after 2 days and 9 (3.2%) started after 3 days. Majority 177 (63.7%) of the respondents replied that they gave ORS solution as much the child could drink, while 84 (30.2%) gave after each loose stool by measuring with cup of coffee as ordered by health care provider.

Most of the mothers 235 (84.5%) preserved prepared ORS for less than 24 h and the rest of mothers used even after 24 h of its preparation.

Regarding practice on zinc supplementation 175 (54.5%) children with diarrhea have given zinc supplementation along with ORS. The duration zinc given was: 46 (26.3%) given for less than 7 days, 73 (41.7%) for 7–9 days, 25 (14.3%) given for 10 days and more, and 31 (17.7%) replied that they stopped to give zinc as soon as diarrhea stopped. All of the children given zinc supplement once a day and majority 260 (90.6%) of them administered zinc by dissolving in water, milk or in ORS solution. More than half 96 (54.9%) of caregivers did not wash their hands before preparing/dissolving zinc for their children. One hundred seventeen (66.9%) of the children received zinc easily or without difficulty and the rest were given zinc forcefully. In general 52% of child caregivers' have good practice toward ORS with zinc depending on 18 questions asked to assess level of their level of knowledge (Table 3).

**Table 3:** Practice of under 5 caregivers about ORS with zinc supplementation for diarrhea management in Burayu town, Oromia, Ethiopia, 2022.

Variables	Response	Frequency	%
Has your child ever suffered from diarrhea (*n* = 422)	Yes	321	76.1
	No	101	23.9
When the child had diarrhea, what do you give to treat diarrhea (*n* = 321)	ORS	102	31.8
	ORS with zinc	175	54.5
	Homemade fluid	109	34
	Usual food	6	1.9
	SSS	25	7.8
	Antibiotics/antidiarrheal	85	26.5
	Solid food alone	1	0.3
Reason for not giving ORS with zinc (*n* = 144)	Did not know where to obtain it	28	19.4
	it is not available	56	38.9
	I couldn't afford	60	41.7
Quantity of water used to prepare ORS (*n* = 278)	1 L/1jug water + 1sacket ORS powder	237	85.3
	2 L water/2 jug + 1sacket ORS powder	19	6.8
	Don't know	9	3.2
	Other	13	4.7
when ORS started	Soon after watery stool noticed	153	55.0
	After 1 day	72	25.9
	After 2 days	44	15.8
	After 3 days	9	3.2
how much ORS given	Up to 2 years: ½‐1 cup after each loose stool, 2 years or older 1–2 cup after each loose stool	84	30.2
	2–3 times a day	156	56.1
	4–5 times a day	36	12.9
	After the passing of every loose stool	60	21.6
Time ORS solution preserved	≤24 h	235	84.5
	>24 h	43	15.5
water used to dilute ORS	Previously boiled and cooled water	126	45.3
	Highland water	122	43.9
	Drinking water for the house (pipe water, river)	30	10.8
Wash hand before ORS preparation	Yes	198	71.2
	No	80	28.8
Did child receive ORS easily?	Yes	183	65.8
	No	95	34.2
When to stop ORS?	When sign of dehydration disappear	141	50.7
	As soon as diarrhea stops	107	38.5
	When the ORS is finished	30	10.8
Given zinc (*n* = 321)?	Yes	175	54.5
	No	146	45.5
Duration zinc given (*n* = 175)	<7 days	46	26.3
	7–10 days	73	41.7
	10–14 days	25	14.3
	Till diarrhea stops	31	17.7
frequency Zn given	Once daily	175	100
How zinc administered	By dissolving (in water, milk, or ORS)	158	90.3
	By swallowing the tablet	17	9.7
hand wash before zinc preparation	Yes	79	45.1
	No	96	54.9
Does your child receive Zn easily?	Yes	117	66.9
	No	58	33.1
Level of practice (mean = 9)	Good practice	167	52.0
	Poor practice	154	48.0

Abbreviation: ORS, oral rehydration salt.

### 3.4 Factors associated with knowledge of mothers/caregivers of under 5 children toward ORS with zinc in Burayu town, Oromia, Ethiopia, 2022

During the binary logistic all variables that had association at *p* < 0.25 were entered to multiple‐logistic regression analysis. Occupation, educational status, income, marital status, and health seeking behavior for diarrhea had significant associations with knowledge of mothers/caregivers toward ORS with zinc at bivariate logistic analysis. In the multivariate logistic regression analysis occupation, educational status, marital status, income, health seeking treatment at government health facility and not taking any health seeking measure for diarrhea had significant association with knowledge of caregiver toward ORS with zinc.

Accordingly, housewives were about 60% times (AOR: 0.407, 95% CI: [0.195, 0.848) less likely to have good knowledge of ORS with zinc for diarrhea management when compared with employees. Mothers/caregivers who had secondary education were about 2.8 times (AOR: 2.75, 95% CI: [1.226, 6.181]) more likely to have good knowledge of ORS with zinc when compared with mothers/caregivers with no formal education. Divorced caregivers were 72.5% times (AOR: 0.275, 95% CI: [0.09, 0.84]) less likely to have good knowledge of ORS with zinc when compared with married. Mother/caregivers with better income (>4000) were more than 5 times (AOR: 5.132, 95% CI: [1.947, 13.524]) more likely to have good knowledge of ORS with zinc when compared with mothers/caregivers with lower income (<2560). Mother/caregivers who did not take any treatment for diarrhea management were 63.1% times (AOR: 0.369, 95% CI: [0.139, 0.979]) less likely to have good knowledge of ORS with zinc for diarrhea management as compared with caregivers who sought treatment for diarrhea management, while, mother/caregivers those sought treatment at government health facility (health centers) were about 4 times (AOR: 3.92, 95% CI: [1.77, 8.67]) more likely to have good knowledge of ORS and zinc as compared with those visited elsewhere (Table 4).

**Table 4:** Factors associated with knowledge of mothers/caregivers of under 5 caregivers toward ORS with zinc in Burayu town, Oromia, Ethiopia, 2022.

Variables	Level of knowledge		COR [95% CI]	*p* Value	AOR [95% CI]	*p* Value
	Good knowledge	Poor knowledge				
Marital status						
Married	237 (61%)	151 (39%)	1	_	1	_
Widowed	3 (33.3%)	6 (66.7%)	0.319 [0.078, 1.293]	0.109	0.355 [0.068, 1.86]	0.220
Divorced	9 (36%)	16 (64%)	0.358 [0.154, 0.832]	0.017	0.275 [0.09, 0.842]	**0.024**
Occupation						
Employees	89 (83.2%)	18 (16.8%)	1	_	1	_
Housewife	75 (41.2%)	107 (58.8%)	0.142 [0.079, 0.255]	0.000	0.407 [0.195, 0.848]	**0.016**
Merchants	34 (52.3%)	31 (47.7%)	0.222 [0.110, 0.448]	0.000	1.059 [0.389, 2.885]	0.911
Others	51 (75%)	17 (25%)	0.607 [0.287, 1.281]	0.190	1.429 [0.55, 3.711]	0.464
Educational status						
No formal education	40 (36%)	71 (64%)	1	_	1	_
Primary school	64 (59.8%)	43 (40.2%)	2.642 [1.528, 4.567]	0.001	3.246 [1.614, 6.53]	**0.001**
Secondary school	38 (59.4%)	26 (40.6%)	2.594 [1.379, 4.879]	0.003	2.753 [1.226, 6.181]	**0.014**
Above secondary school	107 (76.4%)	33 (23.6%)	5.755 [3.321, 9.975]	0.000	6.995 [3.288, 14.883]	**0.000**
Income category						
≤2560	129 (48%)	140 (52%)	1	_	1	_
2561–3200	29 (61.7%)	18 (38.3%)	1.748 [0.927, 3.299]	0.085	1.512 [0.699, 3.271]	0.294
3201–4000	11 (55%)	9 (45%)	1.326 [0.532, 3.305]	0.544	0.467 [0.148, 1.477]	0.195
>4000	80 (93%)	6 (7%)	14.47 [6.102, 34.312]	0.000	5.132 [1.947, 13.524]	**0.001**
Health seeking behavior						
No action taken						
Yes	11 (26.2%)	31 (73.8%)	0.186 [0.09, 0.387]	0.000	0.369 [0.139, 0.979]	**0.045**
No	183 (65.6%)	96 (34.4%)	0.1	_	1	**_**
Taken to government health facility						
Yes	165 (72%)	64 (28%)	5.601 [3.31, 9.478]	0.000	3.92 [1.772, 8.671]	**0.001**
No	29 (31.5%)	63 (68.5%)	1	_	1	_
Taken to private clinics						
Yes	45 (44.1%)	57 (55.9%)	0.371 [0.229, 0.601]	0.000	0.527 [0.256, 1.087]	0.083
No	149 (68%)	70 (32%)	1	_	1	_

*Note*: Bold values are indicate statistically significant *p* value.

Abbreviations: CI, confidence interval; ORS, oral rehydration salt.

### 3.5 Factors associated with practice of mothers/caregivers of under 5 children toward ORS with zinc in Burayu town, Oromia, Ethiopia, 2022

In bivariate logistic analysis, occupation, educational status, sex, marital status, income, level of knowledge, and place sought for treatment were associated with practice of caregivers on ORS with zinc in treatment of diarrhea at *p* < 0.25 and were included into multivariate logistic regression analysis.

In the multivariate logistic regression analysis, occupation, educational status, visiting government health facilities, and knowledge of caregivers about ORS and zinc were significantly associated with practice at *p* < 0.05. Housewives were 91.6% times (AOR: 0.084, 95% CI: [0.029, 0.243]) less likely to have good practice when compared with employees. Mothers with beyond secondary education were more than six times (AOR: 6.26, 95% CI: [1.51, 25.86]) more likely to have good practice when compared with those had no formal education. Mothers/caregivers who visited government health facility (health centers) were about seven times (AOR: 6.885, 95% CI: [2.29, 20.67]) more likely to have good practice of ORS with zinc when compared with who visited elsewhere. Mothers/caregivers who had good knowledge were about 22 times (AOR: 22.14, 95% CI: [8.44, 58.07]) more likely to have good practice of ORS with zinc when compared with mothers who had poor knowledge (Table 5).

**Table 5:** Factors associated with practice of mothers/caregivers of under 5 children toward ORS with zinc in Burayu town, Oromia, Ethiopia, 2022.

Variables	Level of practice		COR [95% CI]	*p* Value	AOR [95% CI]	*p* Value
	Good practice	Poor practice				
Occupation						
Employees	79 (85.9%)	13 (14.1%)	1	‐	1	‐
Housewife	34 (26.4%)	95 (73.6%)	0.059 [0.029, 0.119]	0.000	0.084 [0.029, 0.243]	**0.000**
Merchants	24 (47.1%)	27 (52.9%)	0.146 [0.065, 0.327]	0.000	1.299 [0.277, 6.097]	0.740
Others	30 (61.2%)	19 (38.8%)	0.26 [0.114, 0.591]	0.001	0.538 [0.155, 1.862]	0.328
Sex						
Female	144 (50.5%)	141 (49.5%)	0.58 [0.28, 1.18]	0.134	1.318 [0.376, 4.626]	0.666
Male	13 (36.1%)	23 (63.9%)	1	‐	1	‐
Marital status						
Married	158 (54.5%)	132 (45.5%)	1	‐	1	‐
Widowed	0 (0%)	6 (100%)	0.000	0.999	0.000	999
Divorced	9 (36%)	16 (64%)	0.47 [0.20, 1.098]	0.081	0.538 [0.118, 2.439]	0.421
Educational status						
Illiterate	24 (29.3%)	58 (70.7%)	1	‐	1	**‐**
Primary school	33 (42.3%)	45 (57.7%)	1.772 [0.921, 3.409]	0.086	1.348 [0.398, 4.57]	0.631
Secondary school	25 (55.6%)	20 (44.4%)	3.02 [1.418, 6.436]	0.004	2.324 [0.557, 9.691]	0.247
Above secondary school	85 (73.3%)	31 (26.7%)	6.62 [3.53, 12.42]	0.000	6.26 [1.51, 25.86]	**0.011**
Income						
≤2560	81 (39.1%)	126 (60.9%)	1	‐	1	‐
2561–3200	23 (76.7%)	7 (23.3%)	5.1 [2.09, 12.45]	0.000	2.144 [0.501, 9.173]	0.304
3201–4000	10 (50%)	10 (50%)	1.55 [0.62, 3.903]	0.347	0.664 [0.14, 3.156]	0.606
>4000	53 (82.8%)	11 (17.2%)	7.49 [3.7, 15.2]	0.000	0.360 [0.125, 1.034]	0.058
Health seeking behavior						
Taken to government health facility						
Yes	131 (63.3%)	76 (36.7%)	5.0 [2.9, 8.62]	0.000	6.885 [2.29, 20.67]	**0.001**
No	36 (31.9%)	78 (68.1%)	1	‐	1	‐
Taken to private clinic						
Yes	23 (34.8%)	43 (65.2%)	0.475 [0.294, 0.768]	0.002	2.886 [0.987, 8.437]	0.053
No	144 (56.5%)	111 (43.5%)	1	‐	1	‐
Level of knowledge						
Poor knowledge	10 (7.85%)	119 (92.2%)	1	_	1	**_**
Good knowledge	157 (81.8%)	35 (18.2%)	40.7 [20.43, 81.14]	0.000	22.14 [8.44, 58.07]	**0.000**

*Note*: Bold values are indicate statistically significant *p* value.

Abbreviations: CI, confidence interval; ORS, oral rehydration salt.

## 4 DISCUSSION

This study aimed to assess knowledge, practice and associated factors toward ORS with zinc among under 5 caregivers/mothers of Burayu town. The study revealed that 249 (59%) and 167 (52%) of child's caregivers had good knowledge and good practice of ORS with zinc respectively. Educational status, occupation, marital status, income level and caregivers health seeking behaviors were significantly associated with level of knowledge. Ooccupation, educational status, place sought for treatment, and level of knowledge of ORS with zinc were significantly associated with their practice.

The level of knowledge of management of diarrhea with ORS with zinc in this study is better than what was reported from the study conducted in Goba town, Ethiopia, which indicated 44.8% has good knowledge; however, it is lower than findings from Ginchi town, Ethiopia (63.8%) and for Gojjam town, Ethiopia (63.6%). This difference may be resulted from differences in sample size or health education given for diarrhea management for the respondents.^17^, ^19^

The level of practice in the present study is better than the study conducted in Gojjam town, Ethiopia, which indicated 45.9% of the respondents had good practice. The higher practice on diarrhea management with ORS with zinc in present study might be due to difference in study period as awareness on child care service is improving from day‐to‐day. However, the present result is lower than what was reported from study at conducted at Ginchi town, Ethiopia (52% vs. 59%). The lower practice in present study may be as a result of difference in sample size among the two studies.

Educational status, occupation, marital status, income, and place sought for treatment were significantly associated with caregivers' level of knowledge. Educated mothers/caregivers were more likely to have good knowledge of ORS with zinc when compared with mothers/caregivers with no formal education. As the educational status of mothers/caregivers increases level of knowledge of caregivers about ORS with zinc also increases. This is in line with the study which was conducted in Ginchi town, Ethiopia.^19^ The fact is that highly educated mothers have higher access to information than that of mothers with no formal education and had lower educational status.

In this study, women whose occupation was housewives were less likely to have good knowledge of ORS with zinc for diarrhea management when compared with employees. This finding is consistent with the finding from the study conducted in Iran which indicated a significant association between occupational status and level of knowledge of child caregivers.^27^ The reason may be housewives might not have access to information about ORS with zinc in diarrhea management since they are busy with work overloads.

Divorced caregivers were less likely to have good knowledge of ORS with zinc when compared with married caregivers. The reason may be married caregivers might share information with their husbands.

In addition, mother/caregivers with better income were more likely to have good knowledge of ORS with zinc when compared with those with lower income. This finding is consistent with the study done in Serbo town, Ethiopia, which showed mothers with higher income had good knowledge when compared with those having lower income.^23^ The reason may be those with better income might visit health facilities than caregivers with lower income so that they could get information about ORS with zinc from health facilities or they might access to media or other source of information than those with lower income.

Mother/caregivers who sought treatment at government health facilities were also more likely to have good knowledge of ORS with zinc as compared with who did not visited government health facilities. This is consistent with the study which was conducted in Uganda where mothers/caregivers who visited health centers were more likely to have good knowledge of ORS with zinc when compared with who visited other else. The reason may be due to the fact those who visited government health facilities could heard information about use of ORS with zinc from health care providers and health care providers in government health facilities are access to updated information/training about diarrhea management.

In current study, occupation, educational status, place sought for treatment, and level of knowledge had significant association with practice of mothers/caregivers on ORS with zinc. In this study housewives were less likely to have good practice when compared with employed mothers/caregivers. This finding is similar with the findings from study conducted in Iran which indicated employed mothers had better practice on diarrhea management when compared with housewives.^27^ The reason may be, employed caregivers are most probably educated and they are access to different source of information about child care than housewives.

Mothers/caregivers who attained secondary school and above were more likely to practice ORS with zinc when compared with mothers/caregivers with no formal education. This is consistent with the study which was conducted in Ginchi, Ethiopia, in Fenote Selam, Ethiopia, in Benishangul, Ethiopia, and in Nigeria which showed level of education increases level of practice of diarrhea management.^17^, ^19^, ^26^, ^28^ The fact is that highly educated mothers have higher access of information about ORS with zinc and can practice than mothers with no formal education and had lower educational status.

In addition, place sought for treatment/advice were also associated with practice toward ORS with zinc. Mothers/caregivers who visited government health facility/health centers were more likely to have good practice of ORS with zinc when compared with who visited other else. It is consistent with the study conducted in Debre Birhan Ethiopia and Benishangul Gumuz, Ethiopia, and Uganda.^28^, ^29^, ^30^ This might be due to the fact that health care providers in government facility have access to training which contributes to provision up to date information.

This study also revealed that level of knowledge had positively associated with practice of caregivers toward ORS with zinc. Mothers/caregivers who had good knowledge were more likely to have good practice on ORS with zinc when compared with mothers with poor knowledge. It is similar with the study which was conducted in west Gojjam, Ethiopia, in Debre Birhan, Ethiopia, and in Doba town, Ethiopia, which showed caregivers who had poor knowledge were less likely to have good practice on diarrhea management.^17^, ^30^, ^31^ The fact is that knowledgeable mothers can easily manage diarrhea than mothers who had poor knowledge.

### 4.1 Strength of the study

This study was conducted in a community having advantage than institution‐based studies.

### 4.2 Limitations of the study

The main limitation of this study is that it is limited to cross‐sectional approach, thus unable to formulate a causal association as to how and when the associations are established. Another limitation of this study is that practice of mothers was assessed alone by asking practice question rather than observing while mothers were demonstrating. The study also used only the quantitative data collection method, but it was better if qualitative methods included like observation. It is possible that some caregivers might not remember the details information which might result in recall bias. In addition, shortage of literatures on knowledge and practice of caregivers' on ORS with zinc in diarrhea management was a challenge during writing this thesis.

## 5 CONCLUSION

Based on findings of the study, the researcher has drawn the following conclusions. The more mothers/caregivers are knowledgeable, the more they are active to practice the use of ORS with Zinc. This study revealed that 59% of mothers/caregivers have good knowledge and 52% have good practice toward ORS with zinc. Significant number of mothers/caregivers did not seek any treatment for their children during diarrheal episodes. Educational status, occupation, marital status, place sought for treatment, not taking any treatment action for diarrhea, and income level of child caregivers' had significant association with knowledge of ORS with zinc. Moreover, occupation, educational status, visiting government health facility, and level of knowledge of caregivers had significant association with practice of caregivers on ORS with zinc.

## RECOMMENDATIONS

For health profession

❖ Health care provider should not simply focus on only dispensing either ORS or zinc; they should also spend more time to emphasize on the need of ORS with zinc for the prevention of dehydration due to diarrhea and advice/demonstrate on how to use ORS or zinc.
❖ Activities should also focus as to why, when, and duration it should be used and how correctly it can be used.
❖ There should be continuous in depth/strengthened health education and awareness creation for the mothers/caregivers on appropriate use of ORS with zinc in the management of diarrhea

For community

❖ The community should follow health education given from health professionals to prevent diarrhea and appropriate management of it with ORS and zinc.

For Burayu Health Office

❖ Encourage and facilitates health education program with health professionals.
❖ Encourage mothers/caregivers to involve in health promotion and prevention of diarrhea & dehydration by collaborating with health professionals.

For future researchers

❖ Future researcher also needs to explore the effects of additional variables that were not measured in the current study which can influence mothers/caregivers knowledge and practice toward management of diarrhea by ORS with zinc.
❖ In addition, it is better to deal with qualitative research to explore practice‐related problems in detail.

## AUTHOR CONTRIBUTIONS

**Nadhi A. Duguma**: Conceptualization; investigation; methodology; resources; validation; visualization; writing—original draft; writing—review and editing. **Elias Teferi Bala**: Conceptualization; funding acquisition; investigation; methodology; software; supervision; validation; writing—original draft; writing—review and editing. **Bayisa Abdisa**: Conceptualization; data curation; methodology; software; supervision; validation; visualization; writing—review and editing. **Timketa Adula**: Data curation; resources. **Emiru Adeba**: Formal analysis; resources; software. **Gudina Egata**: Resources; supervision; validation.

## CONFLICT OF INTEREST STATEMENT

The authors declare no conflict of interest.

## TRANSPARENCY STATEMENT

The lead author Elias Teferi Bala affirms that this manuscript is an honest, accurate, and transparent account of the study being reported; that no important aspects of the study have been omitted; and that any discrepancies from the study as planned (and, if relevant, registered) have been explained.

## Data Availability

The data on this study is available and can be shared up on from concerned bodies.

## References

[1] WHO/UNICEF . Clinical Management of Acute Diarrhoeal Disease. UNICEF and WHO; 2004.

[2] WHO/UNICEF . Pneumonia and Diarrhea Progress Report 2020:GAPPD. WHO/UNICEF; 2019–2020.

[3] Prasanna R V , Leo S , Kusneniwar G . Knowledge and attitude of mothers about diarrhea, ors and feeding practices in under‐five children in a rural area of Ranga Reddy, Telangana. J Med Sci Clin Res. 2016;4(10):13201‐13209.

[4] Tilahn TMKBC . ORT on diarrhea: overview of child health problem in Ethiopia. Ethiop J Health Dev. 1995;9(3).

[5] Walker CLF , Bhutta ZA , Bhandari N , et al. Zinc supplementation for the treatment of diarrhea in infants in Pakistan India and Ethiopia. J Pediatr Gastroenterol Nutr. 2006;43(3):357‐363. 16954960 10.1097/01.mpg.0000232018.40907.00

[6] Ayele ED , Tasew H , Mariye T , Teklay G , Alemayhu T , Mesfin F . Zinc utilization and associated factors among under five children having acute diarrhea in Kebri‐Dehar Town, Somali region, Ethiopia 2017. Pathol Lab Med. 2020;4(1):15‐19.

[7] Gebremedin S , Mamo G , Gazahign H , Kung'u J , Adish A . The effectiveness bundling of zinc with oral rehydration salts (ORS) for improving adherence to acute watery diarrhea treatment in Ethiopia: cluster randomised controlled trial. BMC Public Health. 2016;16:457. 27246705 10.1186/s12889-016-3126-6 PMC4888310

[8] Valekar SS , Fernandez K , Chawla PS . Compliance of zinc supplementation by care givers of children suffering from diarrhea. Indian J Community Health. 2014;26(2):41‐137.

[9] Bekele D . *Factors Affecting Use of Zinc Supplementation in Management of Childhood Diarrhea Among Health Workers at Primary Health Care Unit, in Selected Woredas of Bale Zone, South East, Ethiopia*; 2015.

[10] Countdown to 2030 Collaboration . Countdown to 2030: tracking progress towards universal coverage for reproductive, maternal, newborn, and child health. Lancet. 2018;391(10129):1538‐1548. 29395268 10.1016/S0140-6736(18)30104-1

[11] Lazzerini M , Wanzira H . Oral zinc for treating diarrhoea in children. Cochrane Database Syst Rev. 2016;12:005436. 10.1002/14651858.CD005436.pub5 PMC5450879 27996088

[12] WHO . E‐Library of Evidence for Nutrition Action. WHO; 2011.

[13] CSA [Ethiopia] and ICF . 2016 Ethiopia Demographic and Health Survey Key Findings. CSA and ICF; 2017.

[14] CDC . Diarrhea: Common Illness, Global Killer. CDC; 2017.

[15] Jones G , Steketee RW , Black RE , Bhutta ZA , Morris SS . How many child deaths can we prevent this year? Lancet. 2003;362:65‐71. 12853204 10.1016/S0140-6736(03)13811-1

[16] FMOH . Integrated Management of Childhood Illness. FMOH; 2014.

[17] Dereje DAB , Kassa D , Birhanu K , et al. Maternal knowledge and practice towards diarrhoea management in under five children in Fenote Selam Town, West Gojjam Zone, Amhara Regional State, Northwest Ethiopia, 2014. J Infect Dis Ther. 2014;2:182.

[18] Mengistie B , Berhane Y , Worku A . Predictors of ORT use among under five children with diarrhea in Eastern Ethiopia. 2012. 10.1186/1471-2458-12-1029 PMC3560111 23176055

[19] Bekle G . Mothers/caregivers' knowledge, attitude and practice about management of diarrhea and associated factors in under five children in Ginchi town, west Showa, Oromia region, Ethiopia. 2017

[20] Solomon H , Haidar J , Bogale AL . Occurrence of diarrhea and utilization of zinc bundled with ORS among caregivers of children less than five years in Addis Ababa, Ethiopia. J Public Health Epidemiol. 2018;10(9):348‐355.

[21] Burayu Town Municipality . Burayu Town Municipality Annual Report. Burayu Town Municipality; 2021.

[22] Burayu Town Health Office . Burayu Town Health Office Fourth Quarter MCH Report. Burayu Town Health Office; 2020.

[23] Abebe A , Ismael A . Assessment of knowledge, attitude, practice and associated factors on oral rehydration salt (ORS) for diarrhea treatment among mothers of under five children in Serbo Town, South West Ethiopia, 2015: a community‐based cross‐sectional study. J Health Med Nurs. 2015;56:43‐52.

[24] Dawit D , Kumalo E , Yasin Y , Halala Y . Assessment of knowledge, attitude & practice of child care givers towards oral rehydration salt for diarrhea treatment in under 5 children in Wolaita Sodo Town, SNNPR/2016. J Biol Agric Healthcare. 2016;7(4):9‐18.

[25] Acharya NC , Dash Dk , Mohanty MD , et al. Knowledge, attitude and practice of using ORS and household management of childhood diarrhoea. J Nepal Paediatr Soc. 2018;38(2):94‐101.

[26] Gwarzo GD . Mothers' Awareness and Use of Zinc in Under‐Five Children With Diarrhoea in North‐Western Nigeria. Bayero University; 2018.

[27] Khalili M , Mariyam M , Amin Z , et al. Maternal knowledge and practice regarding childhood diarrhea and diet in Zahedan. Iran. 2013;2(1):19‐24.

[28] Misgana HG , Ebessa B , Kassa M . Prevalence of oral rehydration therapy use and associated factors among under five children with diarrhea in Dangure Benishangul region, Ethiopia 2018. BMC Res Notes. 2019;12(1):67. 30700333 10.1186/s13104-019-4078-6 PMC6354353

[29] USAID . Diarrhea Management Knowledge, Attitudes and Practices among Caregivers and Providers in Uganda. USAID; 2012.

[30] Wubetu AD , Engida AS , Yigzaw HB , Mulu GB , et al. Oral rehydration therapy utilization and associated factors in Debre Birhan, Ethiopia, 2020. Pediatric Health Med Ther. 2021;12:251‐258. 34104039 10.2147/PHMT.S312460 PMC8180307

[31] Waktole WK , Gebretsadik GB , Gebremedin GB , Mokonnon TM . Assessment of poor home management practice of diarrhea and associated factors among caregivers of under five children in urban and rural residents of Goba woreda, Ethiopia, comparative cross‐sectional study. Int J Pediatr. 2019;2019:8345245. 31275402 10.1155/2019/8345245 PMC6582835

